# Impact of urban noise pollution on sleep disorders and neurological symptoms

**DOI:** 10.6026/973206300214080

**Published:** 2025-11-15

**Authors:** Lasin Faizal, Anil Kumar Rama Krishnappa, Medha Chakraborty, Shanmukha Koppolu

**Affiliations:** 1 Department of Internal Medicine, Ashford and St Peters Hospital, England, United Kingdom; 2 Department of General Medicine, Sparsh Hospital Yelahanka, Karnataka, India; 3 Department of Internal Medicine, Affiliated Renhe Hospital of China Three Gorges University, China; 4 Department of Trauma and Orthopaedics, Barts NHS Trust, London, England/ UK

**Keywords:** Urban noise, sleep disorders, neurological symptoms, environmental health, cross-sectional study

## Abstract

Urban noise pollution is an emerging public health concern linked to sleep disturbances and neurological dysfunction. This cross- sectional study evaluated the association between environmental noise exposure and the prevalence of sleep and neurological symptoms among 125 urban residents aged 20-55. Noise levels, sleep quality and neurological complaints were assessed using sound meters, the Pittsburgh Sleep Quality Index (PSQI) and clinical interviews. Higher noise exposure correlated with poorer sleep quality and increased reports of headaches, dizziness and irritability. Individuals exposed to noise levels above 65 dB consistently exhibited greater symptom burden, high lighting the neurophysiological impact of chronic urban noise.

## Background:

Urban noise pollution has emerged as a critical public health concern in densely populated cities worldwide [1]. With the rapid pace of urbanization, residents are increasingly exposed to chronic noise from traffic, construction, industrial activity and social sources [2]. While the auditory impact of noise is well-documented, growing evidence suggests that prolonged exposure to high decibel levels also exerts significant non-auditory effects-particularly on sleep and neurological health [3]. Noise exposure can disrupt circadian rhythms, delay sleep onset and reduce overall sleep quality, all of which contribute to physical and mental fatigue [4]. In addition to sleep disturbances, epidemiological and clinical studies indicate that noise acts as a physiological stressor, triggering autonomic arousal and increasing the risk of headaches, irritability, dizziness, cognitive dysfunction and even mood disorders [5]. Despite these associations, much of the existing data are drawn from occupational or experimental studies, with limited cross-sectional data focused on real-world urban residential noise exposure [6]. Therefore, it is of interest to study and compare the impact of urban noise exposure on sleep quality and neurological symptoms among residents in metropolitan environments.

## Materials and Methods:

This cross-sectional study was conducted among 125 adult residents (aged 20-55 years) living in four distinct urban zones of a metropolitan city categorized based on noise intensity: low (<55 dB), moderate (55-65 dB) and high (>65 dB). Participants were selected using stratified random sampling from residential areas adjacent to high-traffic roads, construction zones and quieter suburban neighbourhoods. Eligibility required participants to have lived in their current residence for at least one year and to not be diagnosed with any major neurological or psychiatric disorder. Environmental noise levels were recorded using portable calibrated sound level meters (SLM) at each participant's home over three consecutive days, including both day and night-time readings. The Pittsburgh Sleep Quality Index (PSQI) was used to evaluate subjective sleep quality, with a global PSQI score >5 indicating poor sleep. Neurological symptoms-such as headaches, irritability, dizziness, concentration difficulties and tinnitus-were assessed via structured interviews and symptom checklists developed in accordance with WHO guidelines. Demographic data, lifestyle habits (e.g., caffeine, screen time, alcohol and physical activity) and medical histories were also collected to adjust for potential confounders. Data analysis was performed using SPSS version 26. Descriptive statistics, chi-square tests and one-way ANOVA were used to compare groups. Multivariate logistic regression was applied to determine independent associations between noise exposure levels and health outcomes, adjusting for age, gender, socioeconomic status and lifestyle factors. A p-value of <0.05 was considered statistically significant.

## Results:

Exposure to urban noise pollution was significantly associated with poorer sleep quality and higher prevalence of neurological symptoms. Participants living in high-noise zones (>65 dB) reported significantly greater disturbances in sleep and a higher frequency of symptoms such as headaches, dizziness, irritability and difficulty concentrating. Table 1 (see PDF) presents the demographic characteristics of participants across low, moderate and high noise exposure groups, showing no significant differences in age, gender distribution, employment status, or duration of stay, thereby ensuring balanced baseline characteristics. Table 2 (see PDF) shows sleep quality outcomes, where participants exposed to higher noise levels reported significantly poorer sleep quality, reflected in higher PSQI scores and a greater prevalence of poor sleepers. Table 3 (see PDF) demonstrates the prevalence of neurological symptoms, with headaches, irritability, dizziness and concentration difficulties increasing substantially in the high-noise group. Table 4 (see PDF) presents sleep pattern disturbances, showing that night awakenings, prolonged sleep latency and reduced sleep duration were markedly more common among those exposed to >65 dB. Table 5 (see PDF) highlights the impact of noise on daytime functioning, where fatigue, reduced concentration and memory complaints were considerably higher in high-noise zones. Table 6 (see PDF) presents logistic regression analysis for poor sleep, revealing that moderate and high noise exposures independently predicted poor sleep quality even after adjusting for confounders. Table 7 (see PDF) shows logistic regression for neurological symptoms, indicating that both high noise exposure and poor sleep independently increased the odds of experiencing neurological complaints. Table 8 (see PDF) details the objective noise measurements, showing that high-noise zones consistently recorded elevated decibel levels during both day and night. Table 9 (see PDF) demonstrates the effect of exposure duration, with participants exposed to >65 dB for more than six hours daily reporting the highest prevalence of poor sleep and neurological symptoms. Finally, Table 10 (see PDF) presents protective measures in the high-noise group, showing that interventions such as earplug use, soundproof windows and relocation to quieter rooms were associated with significantly improved sleep outcomes.

## Discussion:

This cross-sectional study highlights a strong association between urban noise pollution and adverse health outcomes particularly sleep disorders and neurological symptoms [7]. The findings align with global literature emphasizing that chronic exposure to environmental noise especially levels exceeding 65 dB can significantly disrupt sleep quality and trigger stress-related neurological complaints [8]. The elevated Pittsburgh Sleep Quality Index (PSQI) scores in moderate and high noise exposure groups reflect impaired sleep architecture, likely driven by repeated arousals and delayed sleep onset [9]. Headaches, dizziness, irritability and concentration difficulties were more prevalent among individuals exposed to persistent urban noise, reinforcing the concept of noise as a physiological stressor [10]. This aligns with known mechanisms: environmental noise activates the hypothalamic-pituitary-adrenal (HPA) axis and sympathetic nervous system, leading to elevated cortisol levels, autonomic imbalance and vascular instability [11]. Additionally, noise-induced sleep fragmentation may impair synaptic recovery and cognitive function, explaining the rise in daytime fatigue and memory issues. The study's strength lies in its real-world data collection across varied urban settings using objective sound level measurements and validated clinical tools [12]. Multivariate analyses confirmed that high noise exposure remained an independent risk factor for poor sleep and neurological symptoms, even after adjusting for confounders such as screen time, alcohol use and gender [13]. Notably, those exposed to noise for over 6 hours per day experienced the most severe health impacts, supporting the idea of a dose-response relationship [14]. Interestingly, behavioural and environmental interventions such as earplug use, window soundproofing, or sleeping in quieter rooms significantly mitigated sleep disturbances among high-noise participants [15]. This suggests that even partial environmental control may offer measurable health benefits in noise-saturated settings [16, 17]. These findings have public health implications for urban design, building regulations and awareness campaigns promoting noise mitigation strategies. However, limitations include the study's cross-sectional nature, restricting causal inference. Recall bias in symptom reporting and lack of objective neurological assessments (e.g., EEG, neuroimaging) may also affect result precision. Longitudinal research is warranted to explore long-term outcomes and potential reversibility of symptoms following noise reduction. Nonetheless, this study underscores the need to recognize environmental noise as a modifiable urban health hazard.

## Conclusion:

Urban noise pollution is significantly associated with increased prevalence of sleep disorders and neurological symptoms. Individuals exposed to high noise levels experienced poorer sleep quality and more frequent headaches, dizziness and irritability. Thus, we show the urgent need for urban noise regulation and personal mitigation strategies to protect neurological and sleep health.

## References

[1] Billings ME (2020). Chest..

[2] Chang VA (2020). Neurocrit Care..

[3] Bani Younis M (2021). Nurs Crit Care..

[4] Yeo AJ (2024). Behav Sleep Med..

[5] Seidler A (2021). Gesundheitswesen..

[6] Smith MG (2022). Environ Health Perspect..

[7] Ebben MR (2021). Sleep Med..

[8] Liu C (2025). Ear Hear..

[9] Li S (2021). BMC Public Health..

[10] Stremler R (2021). JAMA Netw Open..

[11] Gong X (2024). Environ Health Perspect..

[12] Begou P (2021). Environ Sci Pollut Res Int..

[13] Tong H (2023). Environ Res..

[14] Liebich T (2022). Sleep..

[15] Bolognese M (2025). Sci Total Environ..

[16] Rojas-Sánchez OA (2025). Cad Saude Publica..

[17] Sutil DV (2024). Cad Saude Publica..

